# Estimates of Genetic Parameters for Milk, the Occurrence of and Susceptibility to Clinical Lameness and Claw Disorders in Dairy Goats

**DOI:** 10.3390/ani13081374

**Published:** 2023-04-17

**Authors:** Natasha Jaques, Sally-Anne Turner, Emilie Vallée, Cord Heuer, Nicolas Lopez-Villalobos

**Affiliations:** 1 School of Agriculture and Environment, Massey University, Palmerston North 4442, New Zealand; n.lopez-villalobos@massey.ac.nz; 2 Dairy Goat Co-Operative (NZ) Ltd., 18 Gallagher Drive, Melville, Hamilton 3206, New Zealand; 3 EpiCentre, School of Veterinary Science, Private Bag 11-222, Palmerston North 4442, New Zealand

**Keywords:** goat, heritability, genetic correlation, genetic evaluation, lameness, claw disorder, susceptibility, occurrence, milk production

## Abstract

**Simple Summary:**

Lameness and claw disorders are important animal welfare issues in dairy goats, and knowledge is limited. Estimates of genetic parameters for occurrence and susceptibility to clinical lameness and claw disorders and the genetic associations with milk production traits in dairy goats were obtained in this study. These results indicate that a selection index can be developed to select animals resistant or tolerant to lameness and claw disorders.

**Abstract:**

The New Zealand goat industry accesses niche markets for high-value products, mainly formula for infants and young children. This study aimed to estimate the genetic parameters of occurrence and susceptibility of clinical lameness and selected claw disorders and establish their genetic associations with milk production traits. Information on pedigree, lameness, claw disorders, and milk production was collected on three farms between June 2019 and July 2020. The dataset contained 1637 does from 174 sires and 1231 dams. Estimates of genetic and residual (co)variances, heritabilities, and genetic and phenotypic correlations were obtained with uni- and bi-variate animal models. The models included the fixed effects of farm and parity, deviation from the median kidding date as a covariate, and the random effects of animal and residual error. The heritability (h^2^) estimates for lameness occurrence and susceptibility were 0.07 and 0.13, respectively. The h^2^ estimates for claw disorder susceptibilities ranged from 0.02 to 0.23. The genotypic correlations ranged from weak to very strong between lameness and milk production traits (−0.94 to 0.84) and weak to moderate (0.23 to 0.84) between claw disorder and milk production traits.

## 1. Introduction

Dairy goats produced 2% of the total milk produced in New Zealand in 2017 [1]. Under the management of the Dairy Goat Co-operative (NZ) Ltd. (Hamilton, New Zealand), the dairy goat industry in New Zealand became the first commercial producer of dairy goat infant formula and has continued to dominate this niche market internationally [1]. Lameness has been identified as a problem in some dairy goat herds.

Given the potential economic impact, lameness prevention, and control could contribute to farm efficiency, sustainability, and productivity by improving goat welfare. Two New Zealand studies have investigated lameness [2,3]. The first study followed a small cohort of goats during their first two years of life [2]. Deeming et al. [2] reported a small percentage (0 to 8.9%) of lame goats within the group across four locomotion-scoring events after parturition. The second study recorded lameness prevalence between 6.7 and 25.5% in four dairy goat herds [3]. It is unknown what causes lameness in dairy goat herds and whether claw disorders are the predominant reason for lameness. Due to such large variability of lameness prevalence within and between past studies, it is worth exploring risk factors associated with it, including genetics.

Lameness is the impairment of normal locomotion due to pain caused by an injury, disease, or claw disorder [4]. Its etiology can be infectious or non-infectious [5]. Infectious claw diseases are caused by pathogenic bacteria or viruses that colonize on or within their host animal and cause injury to that animal when the conditions are favorable. Non-infectious factors, such as farm management, environment, or genetics, may predispose the goat to lameness.

Selective breeding has been investigated as a long-term option to reduce the prevalence of lameness and claw disorders in dairy cattle and sheep [6,7,8]. Heringstad et al. [8] extensively reviewed the genetics of claw health (infectious and non-infectious claw disorders) in dairy cattle. Overall, claw disorders had low to moderate (0.01 to 0.39) heritabilities across studies. Grouping the claw disorders as one claw health trait or grouping based on etiology resulted in higher estimates for heritabilities. Heritabilities for lameness and claw disorders were low, 0.02 to 0.34. Higher heritability estimates were obtained when lameness was treated as an ordinal trait (1–5 score) than a binary trait (0.08 to 0.15 vs. 0.02 to 0.04, respectively).

A few studies have reported the heritability of claw disorders and lameness in sheep. Studies investigating footrot in 8 to 9 months old lambs reported heritabilities between 0.03 and 0.41 when an occurrence (binary) recording system was used [9,10,11]. Conington et al. [6] studied the prevalence of white line disease and reported heritabilities between 0.09 and 0.33. O’Brien et al. [12] analyzed lameness in various breeds of ewes and lambs and reported an overall heritability for lameness between 0.06 and 0.12. Despite heritability studies in sheep [9,10,11], none have reported genetic or phenotypic correlations between different claw disorder traits and lameness traits.

After an extensive literature search using Web of Science, Scopus, and Google Scholar, only two studies were found that referenced genetics in the occurrence of lameness and claw disorders in dairy goats. Hill et al. [13] reported that the incidence of horn separation was random and, therefore, most likely caused by external factors rather than genetic factors. A second, more recent study reported a moderate to high repeatability of limb problems (arthritis and overgrown claws) and lameness in Damascus, a dairy goat breed in Greece. Repeatability includes the genetic and environmental effects, and low repeatability suggests a greater influence of the temporary environment on the trait of interest [14]. The evidence in sheep and dairy cows suggests that the heritabilities of lameness and claw disorders need to be investigated further in dairy goats.

Exploration of selection against the occurrence and susceptibility of lameness and claw disorders in dairy goats requires estimating genetic parameters, variance and covariances, heritabilities, and genetic and phenotypic correlations between production traits, and occurrence of lameness and claw disorders. Currently, the Dairy Goat Co-operative (NZ) Ltd. (Hamilton, New Zealand). manages the yearly genetic evaluation of dairy goats for lactation yields of milk, fat, protein, lactose, and average somatic cell score (SCS) [15]. Therefore, the objective of this study was to estimate the genetic parameters (genetic variances and covariances, heritability, and genetic and phenotypic correlations) for the occurrence and susceptibility of lameness, claw disorders, and milk production and composition traits in dairy goats.

## 2. Materials and Methods

The animal study protocol was approved by the Ethics Committee of Massey University, New Zealand (MUAEC Protocol 19/51, 29/05/2019).

### 2.1. Data

Data were collected from three dairy goat farms in Waikato, New Zealand, for one production year, from June 2019 to June 2020. All farms volunteered to participate in this study and were shareholders in the Dairy Goat Co-operative (NZ) Ltd. (Hamilton, New Zealand). These farmers had witnessed lameness within their herds and were interested in reducing its prevalence on their farms. Two selected farms had herd sizes around the national average of 750 milking does [16], while one farm was significantly larger, with over 1600 milking does.

The final dataset of seasonal lactation goats contained observations from 1637 does. The pedigree included 174 sires and 1231 dams spanning two generations. Of the does, 83% had records of the sire and the dam, 14% had only information on the dam, and 3% had only information on the sire.

There were a few reference sires between farms, where farm A shared one sire with farm B and three sires with farm C. There was one sire shared between farms B and C. The goat breeds on the three farms consisted mainly of Saanen crosses with other breeds, such as Toggenburg, Alpine, and Nubian [16]. Despite many crosses within the herd, heterosis was not estimated as the data input for the breed information of the animal, sire, and dam and was not precisely recorded within the pedigree.

### 2.2. Milk Trait Characteristics

An orthogonal polynomial model of order 3 was used to predict the total yields of milk, protein, fat, and lactose to 270 days [17]. Aside from calculating total production yields, milk concentrations and ratios were also calculated. The lactation fat, protein, and lactose percentages were calculated by taking the respective lactation yields and dividing these by the total milk yield and then multiplying them by 100. The protein-to-fat ratio (P:F), protein-to-lactose ratio (P:L), and lactose-to-fat plus protein ratio (L:(F + P)) were calculated from the total yields for the respective milk characteristics.

### 2.3. Lameness and Claw Disorder Trait Definitions

Locomotion and claw disorder scoring events were carried out five times for farm A and four times for farms B and C across one lactation from July 2019 to June 2020. Lameness was scored using a 5-point locomotion scale developed by Deeming et al. [18] with minor modifications (Table S1). Briefly, the scores were defined as 0—normal, 1—uneven, 2—mildly lame, 3—moderately lame, and 4—severely lame. A goat was classified as clinically lame if its locomotion was scored as a three or a four. The clinical lameness traits were susceptibility (continuous variable) and occurrence (binomial variable). Clinical lameness susceptibility was the number of times the goat was clinically lame (scored 3 or 4) over the total number of times they were scored between July 2019 and June 2020. The clinical lameness occurrence trait was the diagnosis of clinical lameness (presence or absence) recorded during at least one locomotion scoring event across the production year.

Three claw disorders were selected to focus on; horn separation, rot, and granulomas. Horn separation, a form of white line disease, was a combination of minor and severe hoof horn separation. Minor horn separation was defined as less than 50% of the hoof horn being removed. Severe horn separation was defined as 50% or more of the hoof wall being removed. Severe horn separation was included as a separate trait because it was also associated with clinical lameness (unpublished). Rot was defined as the combination of digital dermatitis and footrot. Granulomas were cherry-like tissue growths protruding from the claw. Each claw disorder was classified as present or absent at each hoof-trimming event. The five claw disorder traits estimated were horn separation susceptibility, severe horn separation susceptibility, rot susceptibility, granuloma susceptibility, and claw disorder occurrence. Claw disorder (horn separation, rot, and granuloma) susceptibility was calculated by the number of times the goat was diagnosed with one of these disorders over the total number of times the hoof trimmer assessed the goat between July 2019 and June 2020. Claw disorder occurrence was the binomial score given (presence or absence) if the goat had at least one case of horn separation or rot or granuloma.

### 2.4. Models to Estimate Genetic Parameters

The genetic parameters (heritability, genetic and phenotypic variances, and covariances) were estimated by Restricted Maximum Likelihood procedures using ASReml version 4 [19]. The milk production traits, lameness, and claw disorder susceptibilities were treated as continuous variables, while lameness and claw disorder occurrence traits were treated as binary variables. This study classified the occurrence as a binary trait and susceptibility as a continuous trait. Both classifications of the traits were assumed to have a normal distribution for both animal and residual variances. In matrix notation, the univariate animal model can be represented as:

**y** = **Xb** + **Za** + **e**

where **y** is the vector of phenotypic observations from measured goats based on one trait, **b** is the vector of fixed effects for goats, **a** is the vector of random animal effects, **e** is the vector of random residual errors, **X** is the incidence matrix for the fixed effects, and **Z** is the incidence matrix combining observations with the random animal additive genetic effects.

Fixed effects in vector **b** include farm, parity, and deviation from the median kidding date as a covariate. The following expectations (E) of the variables were assumed E(**y**) = **Xb**, E(**a**) = **0**, and E(**e**) = **0**. The variances of random effects were assumed as follows: var(**a**) = **A**$\sigma_{a}^{2}$ and var(**e**) = I$\sigma_{e}^{2}$, where $\sigma_{a}^{2}$ is the additive genetic variance, $\sigma_{e}^{2}$ is the random residual variance, **A** the numerator relationship matrix between all animals considered in the data set, and **I** is an identity matrix of order equal to the number of animals with records.

Heritabilities for various traits were estimated with the following equation using variances obtained from the previous univariate analysis:

$$h^{2}=\frac{\sigma_{a}^{2}}{\sigma_{p}^{2}}$$

where $\sigma_{p}^{2}$ is the phenotypic variance calculated as the sum of genetic and environmental variances. Heritability estimates ranging 0–0.10, 0.10–0.60, and 0.60–1.00 were considered as low, moderate, and high, respectively [20]. The bivariate animal model was as follows:

$$\left[\begin{array}{c} y_{1}\\ y_{2} \end{array}\right]=\left[\begin{array}{cc} X_{1} & 0\\ 0 & X_{2} \end{array}\right]\left[\begin{array}{c} b_{1}\\ b_{2} \end{array}\right]+\left[\begin{array}{cc} Z_{1} & 0\\ 0 & Z_{2} \end{array}\right]\left[\begin{array}{c} a_{1}\\ a_{2} \end{array}\right]+\left[\begin{array}{c} e_{1}\\ e_{2} \end{array}\right],$$

where **y_1_** and **y_2_** are the vectors of phenotypic observations from measured goats based on the two traits of interest, **b_1_** and **b_2_** are the vectors of fixed effects, **a_1_** and **a_2_** are the vectors of random animal effects, **e_1_** and **e_2_** are the vectors of random residual errors, **X_1_** and **X_2_** are the incidence matrices for the fixed effects, and **Z_1_** and **Z_2_** are the incidence matrices for the random animal effects. The distribution properties of the variance–covariance elements for traits used in the model for the random animal additive genetic effects and the residual effect are as follows:

$$\mathrm{var}\;\left[\begin{array}{c} a_{1}\\ a_{2} \end{array}\right]=\left[\begin{array}{cc} {A\sigma }_{a1}^{2} & {A\sigma }_{a12}\\ {A\sigma }_{a21} & {A\sigma }_{a2}^{2} \end{array}\right],$$

and

$$\mathrm{var}\;\left[\begin{array}{c} e_{1}\\ e_{2} \end{array}\right]=\left[\begin{array}{cc} {I\sigma }_{e1}^{2} & {I\sigma }_{e12}\\ {I\sigma }_{e12} & {I\sigma }_{e2}^{2} \end{array}\right],$$

where $\sigma_{a1}^{2}$, $\sigma_{a2}^{2}$, and $\sigma_{a12}$ are the additive genetic (co)variance components for the traits of interest, and $\sigma_{e1}^{2}$, $\sigma_{e2}^{2}$, and $\sigma_{e12}$ are the error (co)variance components for the traits of interest.

The following equations defined phenotypic and genotypic correlations:

Phenotypic correlation ($r_{P}$)

$$r_{P}=\frac{\sigma_{p12}}{\left(\sigma_{p1}\times \sigma_{p2}\right)}$$

Genotypic correlation ($r_{G}$)

$$r_{G}=\frac{\sigma_{a12}}{\left(\sigma_{a1}\times \sigma_{a2}\right)}\;,$$

where $\sigma_{p12}$ and $\sigma_{a12}$ are the phenotypic and additive genetic covariance between two traits, $\sigma_{p1}$ and $\sigma_{a1}$ are the phenotypic and additive genetic standard deviations for one trait, and $\sigma_{p2}$ and $\sigma_{a2}$ are the phenotypic and additive genetic standard deviations for the second trait.

## 3. Results

### 3.1. Descriptive Statistics

Descriptive statistics are presented in Table 1. The coefficient of variation (CV) was high for all traits. The CV for production traits ranged from 11.8 to 23.5, while the CV for milk concentration traits ranged from 3.93 to 11.4. The CV for lameness and claw disorders were the highest out of all the traits, ranging from 50 to 338.

### 3.2. Genetic Parameters

Estimates of variance components and heritabilities for the different traits are presented in Table 2. The heritabilities for milk production traits ranged from 0.24 to 0.69. Meanwhile, heritabilities for lameness and claw disorder traits ranged from 0.07 to 0.13 and 0.02 to 0.23, respectively.

Table 3 present the estimates for the genetic and phenotypic correlations. Generally, if there was a relationship, it was due to moderate to very strong positive genetic correlations among the milking traits (0.62 to 0.97). There were a few exceptions. Firstly, a weak positive correlation was observed between somatic cell score and P:F. Secondly, there were strong negative correlations of L:(F + P) with protein percentage and P:L. Lastly, there were moderate negative correlations between P:L and fat percentage, between P:L and milk yield, and between fat yield and P:F.

There were five strong to very strong positive genetic correlations between claw disorder traits and between lameness and claw disorder traits. The correlations were between horn susceptibility and claw disorder occurrence, granuloma susceptibility and rot susceptibility, granuloma susceptibility and lameness occurrence, rot susceptibility and lameness susceptibility, and rot susceptibility and lameness occurrence.

Generally, the correlations between lameness and claw disorder traits and milk traits ranged from near zero to weak, with some exceptions. There was a very strong positive correlation between lameness occurrence and fat percentage. A very strong negative correlation was also found between lameness occurrence and lactose percentage. Rot and granulomas had weak to very strong correlations with milk production yield traits. Horn separation susceptibility and severe horn separation susceptibility had weak and moderate correlations with milk concentration traits, respectively. There were also weak correlations between lactose percentage and granuloma susceptibility and between lactose percentage and claw disorder occurrence.

Generally, the phenotypic correlations between milk production traits were positive and strong or very strong. The two exceptions were for L:(F + P) with protein percentage and P:L, where the strong correlations were negative.

There were only three correlations between the lameness and claw disorder traits. There was one very strong correlation between lameness susceptibility and lameness occurrence, one moderate correlation between horn separation susceptibility and claw disorder occurrence, and one weak correlation between the two horn separation traits. Overall, there were no strong phenotypic correlations between lameness and claw disorder traits with milk traits. There was only a moderate correlation between milk protein percentage and lameness occurrence. There were also weak correlations for lameness occurrence with fat percentage and lactose percentage, between lameness susceptibility and fat yield, between horn separation susceptibility and protein percentage, and between severe horn separation and lactose percentage.

## 4. Discussion

### 4.1. Heritability Estimates

Results from our study indicate that lameness susceptibility, horn separation susceptibility, and rot susceptibility traits are heritable and could be selected against dairy goat breeding programs to improve animal welfare on farms. Our study reported the heritability of lameness susceptibility as a continuous trait rather than a binomial or ordinal trait. Previous studies have reported lameness traits as a binary trait (similar to the occurrence trait in this study) or as an ordinal trait. In dairy cows, the heritability of lameness as a binary trait has ranged from 0.01 to 0.04 using either a linear or threshold model. In contrast, the heritability of lameness as an ordinal trait has ranged from 0.03 to 0.15 [8]. The results in our study were consistent with the study of Weber et al. [21], which also used a 5-point locomotion scale and classified clinical lameness as a locomotion score ≥ 3. Heritability of lameness prevalence was 0.08 and 0.15 when using a linear and threshold model, respectively, similar to our study’s 0.07 and 0.13 for lameness occurrence as a binary trait and lameness susceptibility as a continuous trait, respectively. When modeling binary or ordinal traits, threshold models can account for multiple cases over time, while linear models cannot [8]. Clinical lameness susceptibility had a higher heritability than clinical lameness occurrence. As susceptibility within our study considers multiple cases over a specific time period, it could explain why these heritabilities were similar to the results reported when using the threshold model.

Another factor that may explain the higher heritability estimate for clinical lameness susceptibility than for clinical lameness occurrence is that the calculation of lameness susceptibility includes some degree of the permanent environment effect. Vouraki et al. [14] indicated moderate to high (0.51) between-animal variation of lameness in Damascus dairy goats; however, they were unable to separate the genetic variation from the permanent environmental effects. A permanent environmental effect influences an individual’s performance for a repeated trait. For example, if a doe is recorded as clinically lame in one visit, it could also be identified with clinical lameness in the subsequent visit because she was permanently damaged in the first event. Lameness susceptibility can capture this variation over time, whereas lameness occurrence cannot account for this effect.

Claw disorder susceptibility does not appear to have been studied previously in any species. In our study, horn separation susceptibility had the highest heritability estimate, while the estimate for granuloma susceptibility was the lowest. In the current study, horn separation could be comparable to severe white line disease in dairy cattle, while rot includes digital dermatitis, which has been extensively studied in dairy cattle. The heritability estimates for white line disease and digital dermatitis were higher in this study than those published for dairy cattle. In an extensive review of published heritabilities for claw disorders in dairy cows, traits were classified as binary variables, either presence or absence [8]. White line disease had estimates between 0.01 and 0.10, while digital dermatitis had estimates between 0.01 and 0.09 [8]. These differences in heritability estimates of the two claw disorders could be due to the different definitions of claw disorders. Horn separation is a more severe form of white line disease; therefore, the phenotype would be better defined, easier to identify, and more accurately recorded than white line disease. The more accurately recorded the phenotype is, the higher the heritability [22]. Rot in this study was broadly defined. It encompassed digital dermatitis and footrot because they are similar in their appearance in goats [23]. Due to digital dermatitis appearing differently in cows than goats, the goat’s genetic response may differ from cows. More in line with our study’s estimates were the heritabilities for horn separation and footrot in sheep. Heritability estimates for horn separation have been reported to be between 0.03 and 0.61 [6]. For footrot, estimates were between 0.09 and 0.33 [9,10,11]. These estimates varied depending on the animal’s age (lamb or ewes), breed (Mule or Blackface ewes), number of feet scored, and the type of model used (threshold or linear). One of the traits defined by Nieuwhof et al. [9] had a similar definition to rot susceptibility in this study. Nieuwhof et al. [9] combined and then averaged the footrot scores for Mule ewes across four events. For this trait and using a linear model, they reported a heritability between 0.13 and 0.19 with standard errors between 0.09 and 0.10, which is consistent with the results for rot susceptibility in our study (0.13). Nieuwhof et al. [9] reported that the heritability estimate increased to between 0.21 and 0.23 when the number of affected feet was considered. They also reported that when multiple scores were accounted for over several events, the heritabilities were higher than if only one score was considered [9].

Heritability estimates reported in our study indicate underlying genetic variations for phenotypic traits within dairy goats. Consistent with previous studies, milk production traits in our study showed genetic variation [24]. Our study is the first to report heritability estimates for lameness and claw disorder traits in dairy goats and depicted genetic variation, a novel insight into goat health and breeding. This is in line with a recently published paper that indicated that limb problems and lameness had a moderate to high repeatability in dairy goats; therefore, the potential role of genetics on the occurrence of lameness is indicated [14]. Our study also suggests that lameness and claw disorders should be included in dairy goat breeding programs on commercial dairy goat farms.

### 4.2. Phenotypic Correlations

Currently, clinical lameness and claw disorder traits are not considered in breeding programs for the genetic improvement of dairy goats. One important aspect of introducing them into the breeding program is how the trait is phenotypically and genetically related to other measured traits. The phenotypic correlation between lameness susceptibility and clinical lameness occurrence was strongly positive (0.84), indicating that these two traits are related. However, the genetic correlation was not estimable, possibly due to a low number of observations. The genetic correlations between clinical lameness occurrence and susceptibility and between rot and granuloma susceptibility were positively strong (though relatively high standard errors were also noted to be present), while the phenotypic correlations were weak. Strong genetic correlations indicate that if there is genetic selection against lameness, there will also be genetic selection against rot and granulomas. Weber et al. [21] reported relatively high genetic correlations of 0.60 to 0.95 between lameness and claw disorders (including and excluding digital dermatitis), which was in line with the results of our study. When Weber et al. [21] included digital dermatitis within the claw disorder trait, the genetic correlation with lameness decreased, which is the opposite in our study, where digital dermatitis (included in ‘rot’) had a very strong relationship with clinical lameness. Our study suggests that digital dermatitis in cows may differ from digital dermatitis in dairy goats, which is supported by Groenevelt et al. [25]. The strong genetic correlations estimated in this study between lameness and claw disorders indicate that lameness can be used as an indicator trait for rot and granulomas or vice versa.

Our study determined that there were weak phenotypic correlations between claw disorders. The exception was between all horn separation and the most severe horn separation cases. The weak genetic correlation and strong phenotypic correlation between these two traits indicate that the environment is an important factor in determining the presence of horn separation.

### 4.3. Genotypic Correlations

There were underlying genetic correlations, varying in strength, between the claw disorders. Despite a high standard error, the strongest genetic relationship was between rot and granuloma. The mechanisms behind granuloma development are currently unknown. The granulomas’ low heritability suggests that granulomas occur due to environmental factors. Winter [26] suggested granulomas were related to trimming injuries, although results from our study indicate that this may not be entirely correct. Our results suggest that the relationship between granulomas and hoof-trimming injuries remains unclear. Alternatively, there may be an indirect genetic relationship between rot healing and granulomas developing due to hoof trauma. In addition, horn separation (a form of white line disease) and rot (encompassing digital dermatitis and footrot) susceptibility were very weakly correlated. These were in line with previous research completed in dairy cows on digital dermatitis and white line disease, where genetic correlations ranged from −0.33 to 0.08 [8].

One important aspect of a breeding program for the genetic improvement of dairy goats is how health traits, such as lameness and claw disorders, are phenotypically and genetically related to milk production traits. In our study, clinical lameness susceptibility had a weak negative phenotypic correlation with fat percentage and a weak positive genotypic correlation with P:L ratio. Unlike previous dairy cattle studies, there was little correlation between lameness and milk yield. In our study, the correlation was less than 0.10, while in dairy cattle, it ranged from 0.17 to 0.44 [27,28].

The genetic correlation between the occurrence of clinical lameness and fat percentage was moderately positive (0.75). A reason for this relationship could be the goat’s change in fat percentage in response to metabolic problems, as described for acidosis in dairy cattle. The fat percentage has been linked to negative energy balance and ruminal acidosis in dairy cattle [29]. Negative energy balance has indirectly been associated with the increased risk of lameness through loss of body condition [30,31]. Milk fat depression has been linked with subacute ruminal acidosis in cows [32]. The production of endotoxins resulting from acidosis, which are released into the bloodstream, has been associated with lameness-causing-laminitis [32]. Therefore, in dairy goats, events that may cause a goat to be in a negative energy balance, such as parturition, could increase fat percentage or P:F and indicate a higher risk of lameness in the herd. The results from this study also indicate that there may be some underlying genetic material that could be inherited. Within the breeding goal, selecting goats with higher estimated breeding values for fat percentage could indirectly select goats more susceptible to clinical lameness.

The opposite could be true for lactose concentrations. There was a strong negative genetic correlation between the occurrence of clinical lameness and lactose percentage (−0.94) and subsequently with milk production. It is possible that the control of lactose percentage shares some underlying biological mechanisms relating to the health status and potentially the immune system within dairy goats, as suggested in dairy cows [33,34]. In dairy cows, some studies have reported a negative association between lactose percentage and mastitis [35,36]. While there was not a strong genetic relationship between lameness occurrence and somatic cell score in our study, in dairy cows, one study observed a moderate and weak relationship between lactose yield, mastitis (0.52), and ketosis (0.42), respectively [36]. As Ingvartsen et al. [33] suggested, the effects of genotype, nutrition, time, and management could affect the immune system via the metabolic pathway, which increases the animal’s susceptibility to disease, in this case, lameness. Our study results suggest that lactose percentage may be an important indicator of lameness occurrence within a lactation. This relationship should be further investigated with a larger dataset to validate these results. Other health traits and the relationship with lactose percentage should also be explored in the future.

Claw disorders have weak to moderate genetic correlations with milk production traits. Horn separation traits were negatively correlated with fat percentage and positively correlated with lactose percentage, while rot and granuloma susceptibility were positively correlated with milk yield. This suggests that genetic selection for higher yields of production traits may increase the goat’s genetic susceptibility to rot and granulomas. In contrast, genetic selection for higher concentrations of fat could increase the goat’s genetic susceptibility to horn separation. However, caution should be exercised for this interpretation due to the strength of the correlations. Estimates of genetic correlation between claw disorders and production traits are few in dairy cattle. Depending on the model, Koenig et al. [37] and König et al. [28] reported genetic correlations between digital dermatitis and milk yield of 0.07 to 0.24, which was similar to the values reported in this study (0.31). They also reported correlations of 0.17 to 0.28 between hoof wall disorders and milk yield, which were higher estimates than this study (0.05 to 0.07). Gernand et al. [38] reported weak genetic correlations between digital dermatitis and protein yield, fat and protein percentages (−0.11 to 0.05) which, considering the higher standard error, was still lower than the values calculated in this study (0.08 to 0.41). From the limited number of studies published, the relationship between milk production traits and claw disorder traits in dairy goats is still unclear. Based on dairy cow studies, a negative relationship exists between production traits and claw health. Our study tends to agree with this, although further investigation, with a larger cohort of animals, is needed in goats before conclusions can be made to determine the underlying genetic relationship between claw health and production traits.

### 4.4. Limitations

One of this study’s limitations is the limited number of observations available. Many observations need to be collected on phenotypes of interest to precisely estimate genetic parameters (reducing the standard errors). The current methodology for measuring lameness and claw disorders in dairy goats is time consuming. Locomotion scoring would ideally need to be completed objectively and routinely. Introducing cameras that can record this information automatically would help the farmers immensely. An incentive must also be included for farmers and hoof trimmers to record information on lameness and claw disorders. The farmers would invest in equipment and additional services from the hoof trimmer. Dairy goat hoof trimmers and farmers can only be expected to record this information with reliable technology—preferably designed for dairy goats.

It would also be important to run a multivariate analysis rather than a bivariate analysis to investigate various trait combinations in one model so that covariances can be evaluated simultaneously. This requires more herds with genetic links among the sires. This would only be possible once artificial insemination is widely used within the dairy goat population of New Zealand.

Another approach to estimating heritabilities and variances is using threshold models rather than linear ones. Heritabilities of lameness and claw disorders traits in sheep and dairy cattle have previously been estimated using either threshold or linear models [6,8,9], while genetic and environmental variances can be obtained using linear or liability-threshold models. Threshold models can be better used when the trait of interest is binomial or ordinal and has an underlying continuous phenotype with a low heritability [39]. It is based on the probability of a phenotype being expressed depending on environmental or genetic conditions being met. However, depending on the information available and the trait being used, there may be little difference between the results of the two models [40]. As linear models (using either restricted maximum likelihood or least-squares) are simple and more widely used to estimate genetic parameters, therefore, linear instead of threshold models were chosen to model the estimates in our study [11,41]. However, Heringstad et al. [8] explained that, unlike linear models, threshold models are already adjusted for the time when there are repeated measures of ordinal traits for individuals. Therefore, instead of using the linear model in our study, where we modeled one trait over one lactation at the test-day level (to account for repeated measures over time), the threshold model may have been better for the two binomial traits in our study.

There are two alternatives to the current model that could be used to analyze lameness and claw disorder traits in the future. Firstly, including breed and the heterosis effect within our models. There are significant differences in milk production traits in different dairy goat breeds [24,41], and heterosis has been known to affect milk production traits significantly [15]. In dairy cows and sheep, the breed was a risk factor for developing lameness and claw disorders [6,42]. Therefore, heterosis should be included in the model to capture variation associated with the breed. To do this reliability, the data input of the breeds by the farmers needs to be accurate, which is challenging to achieve when only a pedigree is available.

Secondly, investigate the heritabilities of claw disorder and lameness traits at the test-day level rather than over one lactation. The genetic parameters for milk production traits have been reported to change the lactation [43], and this could also be the case for the genetic parameters of claw disorders and lameness. In dairy cattle, test-day milk yield has been associated with claw disorders [28]. Therefore, it should be established whether claw disorders and/or lameness traits are more heritable at the test-day level rather than at the lactation level.

This study is the first of its kind in dairy goats. It creates the foundation for future research on the genetic parameters of health traits besides the somatic cell score (related to mastitis). The objective would be to eventually include lameness and claw disorder traits in a breeding index to reduce lameness and claw disorder prevalence in herds and improve animal welfare in dairy goats. The economic values and weights need to be estimated before these traits are included in the breeding goal or selection index [44]. The model to estimate these economic values needs to include direct and indirect costs. Indirect costs, such as additional farm labor, should be included, as these costs are realized even when lameness scores are low. In contrast, the direct costs, such as those associated with loss of milk production, are not significant until lameness is severe [45]. Indirect costs may vary depending on the prevalence of lameness and the causes of lameness (which affects the duration) on the farm. The economic weights derived from the influence of other traits, such as milk production traits, claw disorders, and lameness, also need to be included. Finally, using the economic weight and including the desired gains value would establish a selection index for the breeding goal. The desired gains approach would capture costs that may not be monetary, such as animal welfare, and add value to the trait.

Improving claw health through genetics creates permanent gains [8]. Due to the low heritability of health traits, many observations are needed to produce reliable genetic evaluations. Agreeing on the definition of traits is important to be universally understood and have phenotypes accurately recorded.

## 5. Conclusions

This study reported heritability and phenotypic and genetic correlation estimates for claw disorder and lameness traits in dairy goats. The moderate heritability estimates suggest that selecting against clinical lameness, horn separation, and rot susceptibility is possible. There appear to be weak to strong genetic and phenotypic correlations between lameness and claw disorders traits with fat and lactose concentrations in milk. Further investigation into the relationship between these traits is required to determine the underlying biological relationships. If claw disorder and lameness traits are to be included in the breeding goal and selection index for dairy goats, research on the economic impact of these traits is needed in order to assign appropriate economic weights. Before this can be conducted, investing in better technology for dairy goat farmers and hoof trimmers to collect information on lameness and claw disorders is the next step forward.

## Figures and Tables

**Table 1:** Descriptive statistics ^1^ for milk production, lameness, and claw disorder traits in dairy goats from three farms within New Zealand, collected between July 2019 and June 2020.

Trait	N	Average	SD	Min	Max	CV
Lactation length (days)	1637	257	67.5	33.0	350	26.2
Total yields (kg; 270 days)						
Milk	1637	978	228	442	1718	23.3
Fat	1637	31.0	6.99	13.9	54.3	22.6
Protein	1637	30.9	6.76	13.7	53.9	21.9
Lactose	1637	44.4	10.5	19.8	79.4	23.5
Somatic cell score ^2^	1637	9.48	1.12	5.98	13.1	11.8
Milk percentages (%)						
Fat	1637	3.19	0.36	1.91	4.37	11.4
Protein	1637	3.18	0.23	2.47	4.08	7.31
Lactose	1637	4.54	0.18	3.62	5.14	3.93
Milk ratios ^3^						
Protein:fat	1637	1.01	0.11	0.74	1.49	10.8
Protein:lactose	1637	0.70	0.05	0.53	0.96	7.46
Lactose:fat + protein	1637	0.72	0.06	0.56	0.93	7.83
Clinical lameness						
Susceptibility	1625	0.09	0.18	0.00	1.00	208
Occurrence	1637	0.24	0.42	0.00	1.00	180
Claw disorders						
Horn sep. sus. (×10^3^) ^4^	1637	0.43	0.32	0.00	1.00	75.3
Severe horn sep. sus. (×10^3^) ^4^	1637	0.18	0.23	0.00	1.00	127
Rot susceptibility	1637	0.05	0.13	0.00	1.00	244
Granuloma susceptibility	1637	0.04	0.15	0.00	1.00	338
Occurrence ^5^	1637	0.80	0.40	0.00	1.00	50.0

^1^ N = number of records, SD = standard deviation, Min = minimum value, Max = maximum value, CV = coefficient of variation. ^2^ Somatic cell score = average log_2_ (somatic cell count/1000). ^3^ Ratios were calculated using percentages. ^4^ Horn sep. sus. = horn separation susceptibility; Severe horn sep. sus. = Severe horn separation susceptibility. ^5^ Occcurence is the presence of at least one claw disorder or clinical lameness event across the year.

**Table 2:** Estimates of additive genetic and residual variances, heritability, and the corresponding standard errors in parenthesis for milk production, lameness, and claw disorder traits in dairy goats from three farms within New Zealand collected between July 2019 and June 2020 production year.

Traits	Additive Genetic Variance	Residual Variance	Heritability
Total yields (kg; 270 days)			
Milk	8508 (2035)	24,206 (1914)	0.26 (0.06)
Fat	8.93 (1.95)	23.9 (1.84)	0.27 (0.06)
Protein	6.24 (1.53)	19.6 (1.47)	0.24 (0.06)
Lactose	19.3 (4.40)	50.3 (4.10)	0.28 (0.06)
Somatic cell score ^1^	0.28 (0.06)	0.31 (0.07)	0.80 (0.07)
Milk percentages (%)			
Fat (×10^4^)	0.05 (0.01)	0.07 (0.01)	0.56 (0.06)
Protein (×10^4^)	0.03 (0.00)	0.02 (0.00)	0.69 (0.06)
Lactose (×10^4^)	0.02 (0.00)	0.01 (0.00)	0.63 (0.06)
Milk ratios ^2^			
Protein:fat (×10^3^)	3.92 (0.63)	5.57 (0.53)	0.56 (0.06)
Protein:lactose (×10^3^)	1.77 (0.20)	0.86 (0.15)	0.67 (0.06)
Lactose:fat + protein (×10^3^)	1.82 (0.22)	1.20 (0.17)	0.60 (0.06)
Clinical lameness			
Susceptibility (×10^3^)	4.03 (1.76)	26.4 (1.84)	0.13 (0.06)
Occurrence (×10^3^)	0.01 (0.01)	0.15 (0.01)	0.07 (0.05)
Claw disorders			
Horn sep. sus. (×10^3^) ^3^	0.02 (0.00)	0.05 (0.00)	0.23 (0.06)
Severe horn sep. sus. (×10^3^) ^3^	0.01 (0.00)	0.03 (0.00)	0.22 (0.06)
Rot susceptibility (×10^3^)	1.84 (0.71)	12.1 (0.77)	0.13 (0.05)
Granuloma susceptibility (×10^3^)	0.45 (0.82)	21.1 (1.08)	0.02 (0.04)
Occurrence (×10^3^)	0.01 (0.01)	0.11 (0.01)	0.11 (0.05)

^1^ Somatic cell score = average log_2_(somatic cell count/1000). ^2^ Ratios were calculated using percentages. ^3^ Horn sep. sus. = horn separation susceptibility; Severe horn sep. sus. = Severe horn separation susceptibility.

**Table 3:** Estimates of phenotypic (above diagonal) and genetic (below diagonal) correlations ^1^ and standard errors (in parentheses) for milk production, lameness, and claw disorder traits in dairy goats from three farms within New Zealand collected between July 2019 and June 2020 production year.

	Trait	1	2	3	4	5	6	7	8	9
1.	Milk yield		0.81 (0.01)	0.91 (0.00)	0.98 (0.00)	−0.18 (0.03)	0.15 (0.08)	−0.20 (0.05)	0.21 (0.19)	0.00 (0.03)
2.	Fat yield	0.62 (0.09)		0.86 (0.01)	0.83 (0.01)	−0.23 (0.02)	0.31 (0.02)	−0.06 (0.05)	−0.03 (0.05)	−0.46 (0.20)
3.	Protein yield	0.79 (0.05)	0.74 (0.06)		0.92 (0.00)	−0.14 (0.03)	−0.06 (0.05)	0.09 (0.05)	0.16 (0.04)	0.04 (0.26)
4.	Lactose yield	0.94 (0.01)	0.70 (0.07)	-		−0.23 (0.02)	−0.15 (0.05)	−0.18 (0.05)	−0.03 (0.05)	−0.05 (0.03)
5.	Somatic cell score ^2^	0.26 (0.17)	0.17 (−0.01)	−0.01 (0.15)	0.15 (0.33)		−0.10 (0.04)	0.14 (0.26)	0.03 (0.05)	0.21 (0.02)
6.	Fat percentage	-	0.43 (0.11)	−0.05 (0.20)	−0.17 (0.20)	−0.49 (0.24)		−0.12 (0.32)	0.32 (0.06)	−0.14 (0.09)
7.	Protein percentage	−0.21 (0.22)	−0.03 (0.20)	−0.06 (0.25)	−0.18 (0.22)	0.07 (0.11)	0.26 (0.44)		−0.02 (0.08)	0.08 (0.04)
8.	Lactose percentage	-	0.00 (0.11)	0.44 (0.13)	0.00 (0.11)	−0.06 (0.29)	0.28 (0.18)	0.08 (0.18)		−0.23 (0.03)
9.	Protein:fat ratio ^3^	0.07 (0.13)	−0.56 (0.10)	0.13 (0.13)	−0.03 (0.13)	0.35 (0.12)	−0.22 (0.44)	−0.23 (0.12)	−0.31 (0.09)	
10.	Protein:lactose ratio ^3^	−0.53 (0.09)	−0.19 (0.11)	-	−0.42 (0.23)	0.23 (0.10)	−0.59 (0.23)	0.87 (0.02)	−0.27 (0.07)	0.25 (0.09)
11.	Lactose:fat + protein ratio ^3^	0.55 (0.09)	−0.12 (0.12)	-	0.56 (0.08)	−0.05 (0.12)	−0.44 (3.83)	−0.77 (0.03)	−0.01 (0.16)	0.28 (0.09)
12.	Lameness susceptibility	0.09 (0.21)	−0.11 (0.20)	0.16 (0.21)	0.09 (0.21)	0.26 (0.20)	-	0.11 (0.14)	-	0.32 (0.17)
13.	Lameness occurrence	0.07 (0.26)	−0.07 (0.24)	0.14 (0.26)	0.10 (0.25)	0.43 (0.24)	0.75 (0.12)	-	−0.94 (0.03)	0.22 (0.20)
14.	Horn sep. sus. ^4^	0.05 (0.03)	0.10 (0.18)	0.08 (0.18)	0.10 (0.18)	0.07 (0.17)	−0.04 (0.13)	0.37 (0.12)	0.15 (0.13)	0.00 (0.14)
15.	Severe horn sep. sus. ^4^	0.07 (0.18)	0.06 (0.17)	−0.05 (0.18)	0.10 (0.18)	0.06 (0.17)	−0.40 (0.15)	0.34 (0.09)	-	−0.12 (0.15)
16.	Rot susceptibility	0.31 (0.22)	0.14 (0.20)	0.41 (0.22)	0.31 (0.22)	−0.02 (0.20)	0.20 (0.08)	0.08 (0.15)	0.07 (0.15)	0.17 (0.17)
17.	Granuloma susceptibility	0.52 (0.42)	0.63 (0.48)	0.84 (0.49)	0.63 (0.46)	−0.07 (0.35)	0.00 (0.38)	−0.25 (0.27)	0.27 (0.24)	0.07 (0.30)
18.	Claw disorder occurrence	−0.08 (0.23)	0.07 (0.23)	−0.10 (0.23)	0.02 (0.24)	−0.19 (0.22)	0.05 (0.17)	0.18 (0.09)	0.06 (0.27)	−0.22 (0.19)
	**Trait**	**10**	**11**	**12**	**13**	**14**	**15**	**16**	**17**	**18**
1.	Milk yield	−0.42 (0.02)	0.42 (0.02)	−0.05 (0.03)	−0.02 (0.03)	0.05 (0.03)	0.02 (0.03)	0.00 (0.03)	−0.01 (0.02)	−0.05 (0.03)
2.	Fat yield	−0.21 (0.03)	−0.08 (0.03)	−0.45 (0.03)	−0.03 (0.03)	0.07 (0.03)	0.02 (0.03)	0.01 (0.03)	0.00 (0.02)	−0.01 (0.03)
3.	Protein yield	−0.10 (0.02)	0.12 (0.02)	0.07 (0.03)	−0.03 (0.03)	0.07 (0.03)	0.02 (0.03)	0.02 (0.03)	−0.01 (0.02)	−0.03 (0.03)
4.	Lactose yield	−0.14 (0.08)	0.44 (0.02)	−0.05 (0.03)	−0.02 (0.03)	0.06 (0.03)	0.03 (0.03)	0.01 (0.03)	−0.01 (0.02)	−0.04 (0.03)
5.	Somatic cell score ^2^	0.28 (0.02)	−0.14 (0.03)	0.05 (0.03)	0.05 (0.03)	0.03 (0.03)	0.01 (0.03)	−0.02 (0.03)	0.01 (0.03)	−0.03 (0.03)
6.	Fat percentage	0.08 (0.04)	−0.50 (0.23)	−0.19 (0.06)	0.37 (0.08)	0.03 (0.03)	0.10 (0.04)	0.16 (0.04)	−0.03 (0.04)	0.16 (0.05)
7.	Protein percentage	0.85 (0.01)	−0.72 (0.01)	0.02 (0.03)	0.55 (0.21)	0.39 (0.03)	0.20 (0.04)	0.04 (0.03)	0.03 (0.03)	0.06 (0.03)
8.	Lactose percentage	−0.32 (0.03)	−0.18 (0.04)	−0.19 (0.07)	−0.34 (0.04)	0.07 (0.03)	0.39 (0.03)	0.05 (0.26)	0.03 (0.01)	0.29 (0.11)
9.	Protein:fat ratio ^3^	0.25 (0.03)	0.38 (0.02)	−0.01 (0.03)	0.01 (0.03)	−0.02 (0.03)	0.00 (0.03)	0.01 (0.03)	−0.02 (0.03)	−0.06 (0.03)
10.	Protein:lactose ratio ^3^		−0.80 (0.01)	0.01 (0.03)	0.00 (0.03)	−0.01 (0.03)	−0.04 (0.03)	0.01 (0.03)	0.00 (0.03)	0.01 (0.03)
11.	Lactose:fat + protein ratio ^3^	−0.86 (0.02)		−0.02 (0.03)	0.01 (0.03)	0.00 (0.03)	0.03 (0.03)	−0.01 (0.03)	−0.01 (0.03)	−0.05 (0.03)
12.	Lameness susceptibility	0.10 (0.15)	0.09 (0.15)		0.84 (0.07)	0.01 (0.03)	0.06 (0.03)	0.26 (0.02)	0.29 (0.02)	0.03 (0.03)
13.	Lameness occurrence	0.11 (0.18)	0.02 (0.19)	-		0.00 (0.03)	0.06 (0.13)	0.22 (0.02)	0.23 (0.02)	0.05 (0.02)
14.	Horn sep. sus. ^4^	−0.05 (0.13)	0.06 (0.13)	0.18 (0.22)	0.22 (0.28)		0.49 (0.02)	0.21 (0.02)	0.03 (0.02)	0.57 (0.02)
15.	Severe horn sep. sus. ^4^	−0.22 (0.13)	0.14 (0.13)	0.36 (0.21)	0.23 (1.45)	0.63 (0.12)		0.24 (0.02)	0.03 (0.02)	0.25 (0.02)
16.	Rot susceptibility	0.04 (0.15)	0.01 (0.15)	0.70 (0.22)	0.97 (0.25)	0.11 (0.21)	0.02 (0.22)		0.26 (0.02)	0.20 (0.02)
17.	Granuloma susceptibility	0.25 (0.27)	−0.19 (0.28)	0.68 (0.39)	0.95 (0.25)	0.40 (0.40)	0.49 (0.39)	0.92 (0.48)		0.09 (0.02)
18.	Claw disorder occurrence	−0.19 (0.17)	0.09 (0.17)	−0.32 (0.27)	−0.28 (0.31)	0.92 (0.13)	0.45 (0.21)	−0.02 (0.29)	0.02 (0.49)	

Where ‘-’ represents any non-estimable relationship. ^1^ None, weak, moderate, and strong correlations were <|0.3| (green), |0.30| to <|0.50| (blue), |0.50| to <|0.70| (yellow), and |0.70|–|1.00| (red), respectively. ^2^ Somatic cell score = average log_2_(somatic cell count/1000). ^3^ Ratios were calculated using percentages. ^4^ Horn sep. sus. = horn separation susceptibility; Severe horn sep. sus. = Severe horn separation susceptibility.

## Data Availability

Restrictions apply to the availability of these data. Data were obtained from New Zealand Dairy Goat Co-Operative Ltd. and are available from the authors with the permission of the New Zealand Dairy Goat Co-Operative Ltd.

## Supplementary Materials

The following supporting information can be downloaded at: https://www.mdpi.com/article/10.3390/ani13081374/s1, Table S1: Locomotion scoring strategy used to measure lameness in dairy goats (adapted from Deeming et al. [18]).
