# Supplementary material for: Estimates of Genetic Parameters for Milk, the Occurrence of and Susceptibility to Clinical Lameness and Claw Disorders in Dairy Goats
Source: Animals (Basel). 2023 Apr 17;13(8):1374. doi: 10.3390/ani13081374 (PMC10135229; doi:10.3390/ani13081374)
Supplement: Supplementary file 1 [file animals-13-01374-s001.zip › animals-2252720-Supplementary Material.pdf]

animals

MDPI

Supplementary table

Table S1. Locomotion scoring strategy used to measure lameness in dairy goats (adapted from Deeming et al. 2018).

Severity Score	Label	Clinically Lame	Limping	Gait Description	Modifications
0	Normal/not lame	No	No	Moving forward with even strides where hooves track up. Weight-bearing and no apparent head nodding.	
1	Uneven gait	No	Uneven	Moving forward with shorter strides where hooves do not track up. Weight-bearing, and having an absent head nodding, however, have joints that may show stiffness.	
2	Mildly lame	No	Yes	Moving forward with shorter strides where hooves do not track up. One or more legs/feet may be affected. Weight-bearing, and having an absent head nodding, however, may show a mild limp.	
3	Moderately lame	Yes	Yes	Reluctant to move forward and may display a moderate limp. One or more legs could be affected and may display some goose-stepping.	
4	Severely lame	Yes	Yes	Walking on the knees. Refusal to bear any weight on one foot. Severe limping or extreme goose-stepping.	If there is no limp, however, they are walking forward and weight-bearing reluctantly. Multiple feet are involved.
